# Roles, Characteristics, and Analysis of Intrinsically Disordered Proteins: A Minireview

**DOI:** 10.3390/life10120320

**Published:** 2020-11-30

**Authors:** Frederik Lermyte

**Affiliations:** Department of Chemistry, Technical University of Darmstadt, Alarich-Weiss-Straße 4, 64287 Darmstadt, Germany; frederik.lermyte@tu-darmstadt.de

**Keywords:** intrinsically disordered protein, structural biology, biophysics, mass spectrometry

## Abstract

In recent years, there has been a growing understanding that a significant fraction of the eukaryotic proteome is intrinsically disordered, and that these conformationally dynamic proteins play a myriad of vital biological roles in both normal and pathological states. In this review, selected examples of intrinsically disordered proteins are highlighted, with particular attention for a few which are relevant in neurological disorders and in viral infection. Next, the underlying causes for intrinsic disorder are discussed, along with computational methods used to predict whether a given amino acid sequence is likely to adopt a folded or unfolded state in solution. Finally, biophysical methods for the analysis of intrinsically disordered proteins will be discussed, as well as the unique challenges they pose in this context due to their highly dynamic nature.

## 1. Introduction

In the conventional view of molecular biology, proteins fold to adopt a well-defined three-dimensional structure in order to fulfil their biological function. This folding is driven by thermodynamics, so as to maximize interactions such as hydrogen bonds and salt bridges, while shielding hydrophobic residues from the aqueous environment in the cell (in the standard case of a soluble protein). Intrinsically disordered proteins (IDPs) defy this paradigm by adopting a broad range of transient conformations which are similar in free energy, and with no great kinetic barriers to overcome when transitioning between them. Based on the sequence-based prediction of disorder (see Section 3.1), it was estimated in the early 2000s that one-third of eukaryotic proteins have intrinsically disordered regions (IDRs) more than 30 residues long [1,2]. It is important to note in this context that this does not mean that 30% of eukaryotic proteins possess no higher-order structure, as a spectrum exists between fully structured and fully disordered proteins. Many of the proteins possessing IDRs, for instance, have a mostly folded structure with local disorder, or have folded domains which are connected by disordered linkers (e.g., antibodies) [3]. For the sake of simplicity, in the rest of this review the term “IDP” will be used as a catch-all term for proteins that contain significant IDRs, regardless of whether they also have domains with a stable folded structure. Considering the frequent occurrence of IDPs, as well as their important biological roles (both normal and pathological), it is not surprising that this field has garnered significant interest in recent years, and several excellent reviews have been published [4,5,6,7,8,9,10,11,12].

A first class of IDPs is notable not for their normal biological function, but for their pathological role in degenerative amyloid diseases. The most prominent example is alpha-synuclein, associated with Parkinson’s disease, multiple system atrophy, and dementia with Lewy bodies [13,14]. However, prion protein (PrP) and amyloid beta also exhibit a significant disorder [15,16,17,18]. The transition to a more ordered (often beta-sheet rich), aggregation-prone conformational state leads to oligomerization and eventual fibril formation. The underlying process is not fully understood, although there are indications that binding to certain ligands or trace metal ions might play a role [19,20,21,22,23]. A related IDP that is relevant to neurodegeneration is tau. First discovered in 1975 and shown to be essential for microtubule assembly [24], hyperphosphorylated isoforms of this protein are the main constituents of the neurofibrillary tangles observed in the brain of Alzheimer’s disease patients and in other so-called “tauopathies” [25,26].

The archetypal example of a functional IDP is the tumor suppression protein p53, sometimes referred to as the “*Guardian of the Genome*” [27]. By interacting with a myriad of different binding partners, this protein plays several vital roles to maintain genomic stability and promote DNA repair, thereby reducing the frequency of (potentially cancer-causing) mutations [28]. Interestingly, one of the key pathways for this involves another IDP, p21, which in turn is able to bind to and inhibit cyclin-dependent kinase 2, thereby arresting the cell cycle and allowing enough time for damaged DNA to be repaired before it is passed on to the next generation of cells through cell division [29]. Conversely, certain mutant p53 variants are not only unable to facilitate DNA repair, but actively promote tumor progression and metastasis, again through a variety of interactions with other proteins [30]. Another manner in which IDPs interact with the genome is through regulation of gene expression, as silencing of methylated DNA—most often on the cytosine C_5_ carbon of a cytosine/guanine dinucleotide (CpG) —is mediated through several methyl-CpG binding domain (MBD) proteins [31], which contain long IDRs [32,33,34,35]. The most studied of these proteins is MeCP2, the gene for which is located on the X chromosome. Certain mutations in this gene are lethal in males and linked with Rett syndrome, a progressive neurodevelopmental disorder, in females [36].

Paradoxically, IDPs play a vital role in ensuring that other proteins are folded correctly, since many chaperone proteins exhibit some level of intrinsic disorder [37]. For example, in the bacterial GroEL-GroES complex—a 21-mer composed of 14 GroEL and 7 GroES monomers—a disordered 23-residue C-terminal portion of GroEL faces the central cavity of the complex in which folding occurs. Removing this disordered tail has been shown to lead to a dramatic deterioration in the chaperone function [38]. Similarly, IDRs are found in human small heat shock proteins, produced as part of the cellular stress response, such as Hsp22 and αB-crystallin [39,40]. The examples provided here serve to illustrate the ubiquitous nature and critical biological importance of IDPs. In the rest of this review, the underlying causes for intrinsic disorder will be discussed, both from a physicochemical perspective (i.e., the thermodynamic factors that cause a protein to not adopt a well-defined structure in the solution), as well as an evolutionary one (i.e., how their intrinsic disorder allows these proteins to fulfil their biological role). Finally, a selection of computational, as well as experimental methods for investigating IDPs will be considered.

## 2. General Characteristics of IDPs

### 2.1. Physicochemical (Sequence) Characteristics

As mentioned in the Introduction, protein folding is driven by thermodynamics. Hydrogen bonding, dipole interactions, and salt bridges all result in enthalpic stabilization; however, it should be noted that similarly favorable interactions are generally possible with water molecules in the environment. Meanwhile, the entropy of the protein backbone is reduced upon restriction of conformational freedom, but for most folded proteins, this is offset by the ability to shield hydrophobic side chains in the interior of the protein structure, which increases the entropy of the surrounding water molecules. Given these factors, it should come as no surprise that IDPs are generally characterized by a small number of hydrophobic residues, and a large number of charged residues [41,42].

Another common characteristic of IDPs is that they tend to have an uncompensated net charge at physiological pH. Two important metrics used to assess the (dis)ordered nature of protein sequences are the fraction of charged residues (FCR) and net charge per residue (NCPR) [42,43]. FCR is simply the number of residues that have a net charge—either positive or negative—at pH 7 (i.e., D, E, K, R) divided by the total number of amino acid residues in the sequence. NCPR is the net charge (i.e., the number of positively charged residues minus the number of negatively charged residues) divided by the total number of residues. As stated previously, charged side chains are to some extent a double-edged sword in the context of protein folding—on the one hand, they allow for favorable interactions with water molecules (which favors unfolding), but salt bridges between oppositely charged side chains can also stabilize a folded structure. For this reason, Das and Pappu developed a more sophisticated metric that reflects the linear sequence distribution of positively and negatively charged residues [44]. This charge mixing metric is essentially calculated by evaluating the FCR and NCPR not just for the entire protein, but also across a sliding window of five or six residues (the final step in the calculation involves averaging the results for both window sizes). In this manner, the degree of charge asymmetry in all of these small sub-sequences is calculated, summed, and normalized to the extreme case of a sequence with the same length, FCR, and NCPR, but with perfect charge separation, i.e., all charged residues consolidated into a positive and negative “block”, positioned at both sequence termini. In this manner, a parameter *κ* is obtained, which varies from 0 for perfectly mixed sequences, to 1 for sequences where positive and negative charges are perfectly segregated. Note that artefacts can occur in this calculation, for example, if there are very few charged residues, which can lead to calculated *κ* values greater than 1. Sequences with low *κ* values are more likely to be disordered, as many transient interactions with nearby oppositely charged residues are possible, whereas sequences with oppositely charged blocks are more likely to form hairpin-like conformations where these blocks can interact [44].

**Figure 1:**
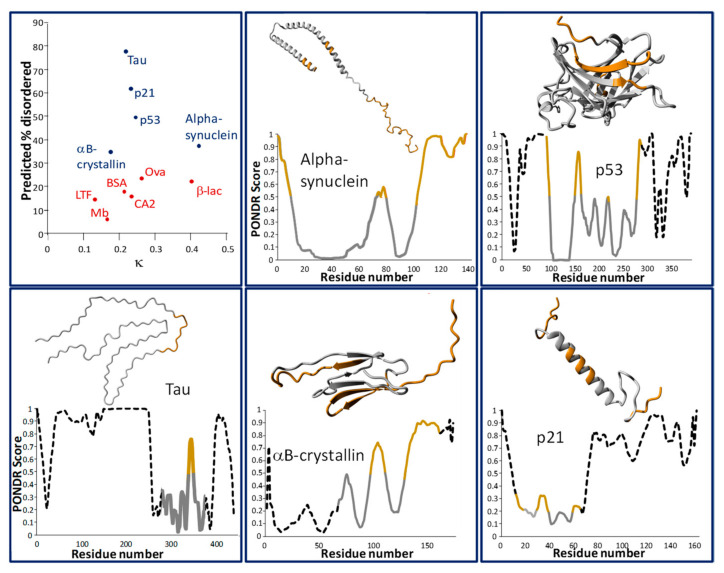
(**Top-left**) Predicted fraction of residues in disordered regions versus charge mixing parameter *κ* for five intrinsically disordered (Tau, p21, p53, αB-crystallin, and alpha-synuclein) and six globular (myoglobin, lactotransferritin, carbonic anhydrase 2, bovine serum albumin, chicken ovalbumin, and bovine β-lactoglobulin) proteins. (**Other panels**) show results for the five intrinsically disordered proteins (IDPs) in detail, displaying (partial, except for alpha-synuclein) experimentally obtained structures and the PONDR (Predictor of Natural Disordered Regions) score per residue. Note that for several of these structures, a complex with interacting proteins or ligands (not shown) was analyzed rather than the monomeric IDP, resulting in a more ordered structure than might be expected. Regions predicted to be disordered by the PONDR algorithm (score > 0.5) are shown in gold in both the graphs and structures, except for p21, where a lower cut-off (0.2) was used to highlight the local maxima due to none of the parts of the sequence which are included in the experimentally observed structure scoring as “disordered”. PONDR scores for regions which are not part of the experimentally obtained structure are shown as a black dotted line in the graphs, regardless of their value. Protein Data Bank (PDB) identifiers for the structures are as follows—tau: 6TJO (441-residue isoform; structure obtained by cryo-EM of filaments); p21: 6P8H (XRD; measured as part of a complex with cyclin-dependent kinase 4 and cyclin D1); p53: 1TUP (XRD; measured as part of a complex with DNA); aB-crystallin: 3L1G (XRD); αSN: 1XQ8 (solution NMR; micelle-bound). Unless otherwise noted, the unmodified human sequence variant of these proteins was used in the calculations. Protein structure visualizations were generated using YASARA View [48].

In Figure 1, two relevant parameters for five of the examples of IDPs discussed in the Section 1 are shown in a 2D plot (top-left panel; blue dots). On the vertical axis, the fraction of residues incorporated in disordered regions is shown, as predicted by the PONDR-VLXT algorithm (see Section 3.1) [45]. On the horizontal axis, the charge mixing parameter *κ* is shown. For reference, these two parameters were also calculated for six globular proteins (data points in red). As expected, most of the proteins with significant disordered regions displayed in Figure 1 indeed have *κ* values below 0.3, with the exception being alpha-synuclein (*κ* = 0.42) which has a positively charged N terminus and a negatively charged C terminus, which are known to interact with one another to form transient tertiary structures [46,47]. Five of the six globular proteins, however, also have *κ* values below 0.3 (range: 0.13–0.40), indicating that while this parameter is useful for comparing permutants of a single sequence, it alone is not sufficient for classifying a protein (region) as ordered or disordered. The more sophisticated prediction method (PONDR), however, was able to accurately classify the globular proteins as possessing less disorder than the IDPs in this small data set. In the other panels of Figure 1, the PONDR score per residue is displayed for each of the five IDPs considered, with regions scoring high (corresponding to a prediction of a more disordered region) displayed in red. Above the graphs are (partial) structures for these proteins, obtained through cryo-electron microscopy (EM), X-ray diffraction (XRD), or nuclear magnetic resonance (NMR). In most cases, structures for the full-length proteins are not available, illustrating the analytical challenge that IDPs pose (discussed further in Section 3.2). In these cases, sequence regions which are not part of the crystal structure are displayed as a dotted black, rather than solid grey, line in the graph.

**Figure 2:**
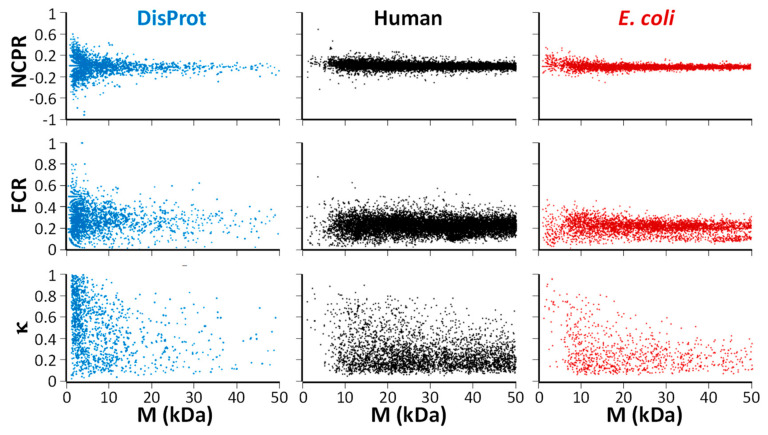
Net charge per residue (NCPR; **first row**), fraction of charged residues (FCR; **second row**), and *κ* (**third row**) for proteins with masses up to 50 kDa for (**first column**; in blue) sequences in the DisProt database (all intrinsically disordered), (**second column**; in black) human proteins (ca. 30% disordered), and (**third column**; in red) *E. coli* (<5% disordered).

To more systematically investigate the lack of clear correlation between the *κ* value and degree of disorder seen for the small data set in Figure 1, three important parameters—NCPR, FCR, and *κ*–were calculated for (1) all sequences in the DisProt database of disordered proteins (release 2020_06) [49,50,51], (2) all human proteins in the SwissProt database, and (3) all *E. coli* (strain K12) proteins in SwissProt. As discussed earlier, approximately 30% of human proteins have significant disordered regions, whereas for *E. coli*, this is less than 5% [2]. Of course, 100% of the sequences in DisProt are intrinsically disordered. The result of this calculation is shown in Figure 2. In this figure, each protein with a mass up to 50 kDa (6656 Disprot sequences, 20,342 human proteins, and 4518 *E. coli* proteins) is represented by a data point in three ((parameter) vs. mass) plots, where the three aforementioned parameters are shown in the first, second, and third row, respectively. Interestingly, while the disordered sequences seem to have a greater diversity of NCPR and FCR values—especially at low masses—it is clear from comparing the three columns that caution is warranted when using only these simple parameters for protein classification.

Of note is recent work by Mittag and Pappu et al., in which it was found that, in addition to the patterning of charged residues, patterning of aromatic residues in intrinsically disordered prion-like domains determines their tendency to undergo a liquid-liquid phase separation (LLPS) [52]. This process is increasingly understood to be important for the formation of membraneless organelles such as nucleoli and stress granules [53]. However, it may also play a role in disease, such as neurodegenerative disorders. For example, it has been shown that amyotrophic lateral sclerosis-related mutations in TDP-43 lead to a reduced capacity to undergo LLPS [54]. Conversely, the concentration of proteins such as tau, alpha-synuclein, or huntingtin in phase-separated droplets may provide favorable nucleation conditions for potentially pathological fibril formation [55]. Combining NMR, small-angle X-ray scattering (SAXS), and all-atom simulations, a “stickers-and-spacers” model was developed, in which aromatic residues are the “stickers” that drive intra- and intermolecular interactions. A parameter *Ω_aro_* was defined to quantify how uniformly aromatic residues are distributed along the sequence and it was found that, for the prion-like domain of heterogeneous nuclear ribonucleoprotein A1, the aromatic residues are more uniformly spaced (lower *Ω_aro_*) than expected by chance (*p* < 0.0001). Other proteins known to undergo LLPS were shown to have similarly high degrees of uniformity in their distribution of aromatic residues along the sequence. Higher values of *Ω_aro_* for these proteins were associated with aggregation, rather than phase separation. Thermodynamically, this is explained by the energetic stabilization resulting from the “clumping” of stretches rich in aromatic residues being sufficient to overcome the stabilization provided by solubilization of the more hydrophilic spacers.

Given the importance of charged residues in determining whether a protein adopts a well-defined structure, it can be expected that post-translational modifications—particularly ones such as phosphorylation, which converts a neutral residue to a negatively charged one—could have a significant conformational effect [13]. In one recent example, Mittag and Pappu et al. studied the effect of phosphorylation on the intrinsically disordered region of the *S. cerevisiae* transcription factor Ash1 (residues 420–500) [56]. Remarkably, of the 81 residues in this protein, 10 are possible phosphorylation sites, 17 are charged (16 positive, one negative; FCR = 0.21, NCPR = 0.19, *κ* = 0.79), and 12 are prolines. Using SAXS, multi-dimensional NMR, and all-atom Monte Carlo simulations, it was found that the global conformational properties of Ash1^420−500^ did not change upon multiple phosphorylation. It was concluded that the conformational behavior in this case could be rationalized by the linear sequence patterning of prolines and charged residues. Note that, while the value for *κ* is very high, this is due to the fact that nearly all (94%) of the charged residues in the sequence are positive, which illustrates a limitation of the use of this parameter. Interestingly, enhanced R_2_ relaxation rates were observed in NMR after phosphorylation, indicating a less dynamic central region (around residues 450–460). The experimental methods used in this work unfortunately could not probe the transient interactions that led to this less dynamic behavior in detail.

One factor which is often neglected is the fact that the cell is a much more crowded environment than the in vitro samples typically used in biophysical or structural biology studies, and this can affect the conformational dynamics of proteins. In this context, the possibility to perform in-cell NMR must be mentioned, as discussed further in Section 3.2. Schuler et al. have explored the dynamics of intrinsically disordered proteins (C- and N-terminal segments of prothymosin α, activator for thyroid hormones and retinoid receptors (ACTR), and the N-terminal domain of the HIV-1 integrase) in the presence of high volume fractions (up to more than 30%) of polyethylene glycol (PEG) of different average lengths (PEG200 up to PEG35000) [57,58]. Using single-molecule Förster resonance energy transfer (FRET) spectroscopy, they found that energy transfer efficiency increased—indicating compaction of the conformation—upon either an increase in the PEG volume fraction, or chain length. They were able to explain these findings quantitatively using a modified Flory-Huggins theory of polymer solution. This compaction in an environment that more faithfully mimics that of the cell has important implications for the in vivo behavior of IDPs, including a reduced capture radius for binding targets, and an increased diffusion coefficient. It should be noted, however, that many transient interactions occur *in cellula*, both specific and non-specific, and for a truly realistic modelling of this environment, an excluded volume effect alone is insufficient [59].

### 2.2. Evolutionary Characteristics

Having considered the underlying physicochemical causes of why an IDP does not adopt a unique 3D structure, it is useful to also consider the functional reason. The primary benefit of the conformational flexibility of IDPs is generally that it enables a certain level of binding promiscuity—indeed many IDPs are “hubs” in interaction networks and are able to bind/interact with several different targets through an induced fit/conformational selection mechanism, rather than the interactions of more rigid proteins, which resemble more closely the traditional “lock-and-key” mechanism [9,10]. Chaperone proteins provide one example of this, as they need to ensure the proper folding of a variety of substrate proteins. However, the archetypal example of a protein with an extremely broad range of binding partners is p53, as mentioned in the Introduction. This protein displays enormous binding promiscuity, as reviewed in 2016 by Uversky, who showed that the interactome of p53 comprises hundreds of partners [28]. The reason for this exceptional promiscuity—even compared to other IDPs—is that p53 occurs in several different proteoforms: Not only is it able to form homotetramers, but alternative splicing leads to nine relatively common isoforms, and 60 of the 393 residues—many of which are located in intrinsically disordered regions—in this protein can be post-translationally modified. By these mechanisms, hundreds of p53 proteoforms can be produced for specific functions.

Other than the role of IDPs as interaction hubs, a somewhat less commonly cited possibility, which is of significant current interest, is one proposed by Uversky et al. in which intrinsically disordered regions in virus capsids are related to transmission pathways. Specifically, they proposed that capsids with low levels of disorder form a robust protective shell for virions, allowing them to remain infectious outside the body [60,61,62,63]. Conversely, shells with higher levels of disorder were suggested to be characteristic of viruses that rely on airborne transmission. In early 2020, they applied this model to the nucleocapsid (N) and membrane (M) proteins of a range of human and animal coronaviruses, including SARS-CoV-2, responsible for the ongoing Covid-19 pandemic [64,65]. Based on this analysis, they concluded that SARS-CoV-2 spreads through both respiratory and faecal-oral pathways, and that virions are sufficiently robust that an infected body is likely to shed large numbers of infectious particles. While current mitigation strategies are primarily focused on preventing airborne transmission through aerosols [66], strategies such as regular handwashing are still promoted throughout the world to prevent fomite-based transmission [67]. It is likely that both pathways play a role to some extent in real-life scenarios, and it will be interesting to see how well this prediction holds up as more data become available over time.

**Figure 3:**
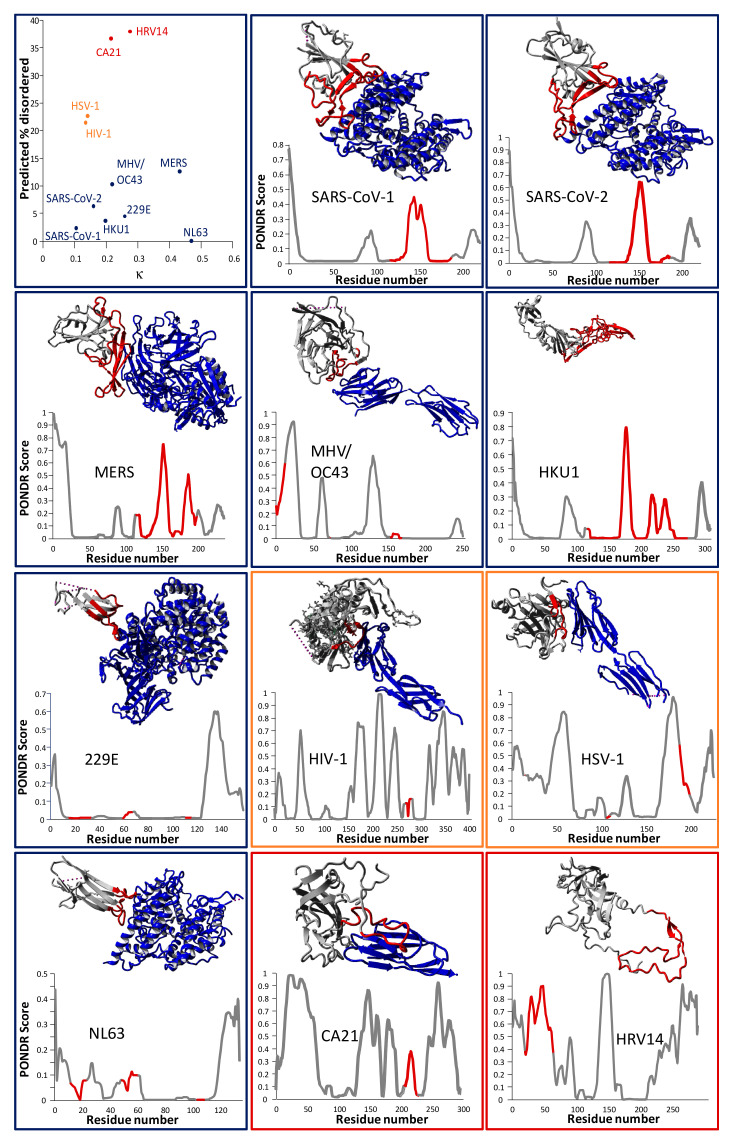
(**Top-left**) The same analysis as in Figure 1 was performed for the key cell entry proteins of 11 human viruses, i.e., all seven human coronaviruses, HIV-1, HSV-1, CA21, and HRV14. Interestingly, the coronavirus spike proteins (in blue; targeting various receptors) are less disordered overall than the other four viral proteins, but span a range of *κ* values, while HIV-1/HSV-1 (orange; targeting CD4 [68] and nectin-1 [69], respectively) and CA21/HRV14 (red; both targeting ICAM-1) [70,71] seem to cluster together in the 2D plot. In the (**other panels**), crystal structures of the viral proteins are shown in the complex with their receptors (except for HCoV-HKU1 and HRV14, for which no structure of this complex was readily available), together with PONDR scores per residue. Border colors of these frames match the dots in the top-left panel. The receptor-binding domain (RBD) is shown in red in both the graphs and the structures. No crystal structure was readily available for the HCoV-OC43 spike protein. Therefore, the spike protein of the closely related (71% genome identity) murine hepatitis virus A59 (MHV) is shown [72]. PDB identifiers for the structures are as follows—SARS-CoV-1: 2AJF (XRD); SARS-CoV-2: 6LZG (XRD); CA21: 1Z7Z (cryo-EM); HCoV-NL63: 3KBH (XRD); MHV: 3R4D (XRD); HSV-1: 3SKU (XRD); MERS-CoV: 4L72 (XRD); HCoV-HKU1: 5KWB (XRD); HCoV-229E: 6ATK (XRD); HRV14: 1HRV (XRD); HIV-1: 1GC1 (XRD). Grey dashed lines present in some of the structures represent short stretches of the sequence which were not resolved. Protein structure visualizations were generated using YASARA View [48].

Viruses also provide insight into the selection pressure for disordered regions in proteins which are able to engage multiple targets. An ability to engage the human analogue of the protein used for cell entry in the normal animal reservoir of a virus allows initial entry into the human population, and a certain degree of disorder could be expected to be beneficial in this context. Calculating the charge mixing parameter *κ* and performing sequence analysis with the PONDR-VLXT algorithm [45] for the key cell entry protein of a range of viruses (spike protein of the seven known human coronaviruses, envelope glycoprotein 120 of human immunodeficiency virus 1, glycoprotein D of herpes simplex virus 1, capsid protein VP1 of human coxsackievirus A21, and viral protein 1 of human rhinovirus 14) reveals evidence of significant levels of disorder in these proteins (see Figure 3). With the exception of MERS-CoV and HCoV-NL63, all 11 viral proteins in this data set had *κ* values below 0.3. The PONDR algorithm predicted that intrinsically disordered regions comprise up to 35% (in HRV14 and CA21) of these sequences. Interestingly, while coronavirus spike proteins show less overall disorder according to the PONDR analysis than the other four examples, for the betacoronaviruses (SARS-CoV-1, SARS-CoV-2, MERS-CoV, HCoV-OC43, and HCoV-HKU1) the receptor-binding domain (RBD) comprises the most disordered sequence region.

Understanding the conformational dynamics of these proteins may have important public health implications—not only is this manuscript being written during the Covid-19 pandemic (caused by SARS-CoV-2), but SARS-CoV-1 caused a major outbreak in 2003 [73]. MERS-CoV, while rare, is highly lethal [73], and genomic analysis of the commonly occurring HCoV-OC43 indicates that bovine-to-human zoonosis occurred around 1890, suggesting that the 1889–1890 pandemic—often attributed to H2N2 influenza—was caused by the first introduction of this virus to an immunologically naive human population [74]. The importance of understanding the structure and dynamics of the spike protein is also highlighted by neutralizing spike protein-reactive antibodies having been shown to persist in recovered Covid-19 patients on a timescale of at least seven months, and by several ongoing vaccine development projects which target this protein [75,76,77]. The HIV/AIDS pandemic has been ongoing since the 1980s, and continues to claim hundreds of thousands of lives each year [78].

## 3. Analysis of IDPs

### 3.1. Computational Methods for Sequence-Based Prediction of Disorder

There is an obvious need for algorithms that can predict, based on a protein sequence, whether that protein is likely to be ordered or disordered under physiological conditions, and in the latter case, which regions will exhibit the greatest degree of disorder. Simple, global physicochemical parameters such as FCR, NCPR, and *κ* are trivial to calculate, but dozens of computational algorithms have been developed over the years. Many of these were recently reviewed by Liu et al. [79]. Broadly speaking, these algorithms can be classified based on their complexity. The simplest ones use physicochemical properties, discussed in Section 2.1, to predict the disorder from first principles. An example of this is FoldIndex [80], which works by evaluating the mean net charge and hydrophobicity as defined by Uversky et al. [41] across a sliding window in order to identify “regional” folding propensities throughout a given sequence. IUPred [81,82,83] is designed along somewhat different principles and predicts disorder based on pairwise intramolecular interaction energies between amino acid residues in a sequence. The assumption in this case is that if the stabilization from such interactions is insufficient to offset the reduction in entropy that results from folding, then the protein is likely to adopt a disordered state. GlobPlot [84] is another relatively straightforward algorithm, and defines a propensity for each of the 20 common amino acid residues to be in an ordered or disordered sequence region. This is again evaluated across a sliding window. TopIDP [85] is built following similar principles and ranks amino acid residues as (W, F, Y, I, M, L, V, N, C, T, A, G, R, D, H, Q, K, S, E, P) from order- to disorder-promoting.

A more advanced class of algorithms uses machine learning to distinguish ordered from disordered sequences. While these have the benefit of being based on training sets containing empirical data, results are not as straightforward to rationalize as for the physicochemical methods. To improve the chances of obtaining accurate predictions from these somewhat opaque algorithms, “meta” methods make up a third category and incorporate several of the predictors from the first two categories and “fuse” these to form a consensus prediction. The PONDR series of algorithms utilizes a neural network to predict disorder. Interestingly, during the development of these algorithms, the authors found that slightly different versions of the algorithm yielded more accurate disorder predictions in sequences of different chain lengths [45,86,87]. Other popular machine-learning algorithms for disorder prediction include DISOPRED [88] and DISOPRED2 [2], developed at University College London, and DisEMBL [89], developed at the European Molecular Biology Laboratory. Very recently, a new neural network-based method called ODiNPred was introduced, with initial results indicting that it may be able to outperform many older algorithms in a head-to-head comparison. The PONDR family of algorithms also includes a meta method, i.e., PONDR-FIT [90]. This combines machine-learning algorithms PONDR-VLXT, PONDR-VL3, and PONDR-VSL2 with physicochemical algorithms IUPred, FoldIndex, and TopIDP. After evaluating a sequence with these six algorithms in parallel, a consensus prediction is generated across a sliding window. The integration of these six outputs is achieved through another neural network, which was shown to result in more informative consensus outputs than simple vote-counting or a linear combination of scores from independent algorithms. DISOPRED3 [91] is a meta-predictor which was developed using similar principles, and builds on the DISOPRED2 algorithm.

Finally, moving beyond the general prediction of order or disorder, computational methods—specifically, molecular dynamics simulations—can be used to supplement experimental results and obtain more detailed structural insights. This has the benefit of allowing the probing of systems and timescales which are not (easily) experimentally accessible, and can therefore be a valuable tool in the study of IDPs [92,93,94]. Care must be taken in such studies, however, as it has been shown that results of such MD studies can depend strongly on how the simulation was set up. Specifically, the choice of force field can have a strong effect on the outputs, as shown by Rauscher et al. [95]. In their work, these authors showed that the CHARM 22* force field was the best option overall, based on a comparison to SAXS and NMR data for a disordered arginine/serine peptide [96]; however, it remains to be seen to what extent this result can be generalized to other IDPs.

### 3.2. Experimental Methods for Structural Characterization of IDPs

Selected methods of particular importance are highlighted in this section. For a more comprehensive overview, we refer to the excellent recent review by Longhi et al. [97]. The two most commonly used methods in structural biology are XRD and NMR. XRD requires that samples are in a crystalline state, i.e., that the molecules in the sample adopt a repeating pattern of well-defined conformations. This is complicated due to the tendency of IDPs to dynamically sample a large conformational space, and although it is possible to obtain crystal structures from pure IDPs [98], crystals are often derived from either smaller fragments that tend to adopt a well-defined structure, or from noncovalent complexes with binding partners that induce folding [99,100]. A method that circumvents the need for large monocrystals is micro-electron diffraction (microED), pioneered by Eisenberg and Gonen [101,102]. In this cryo-EM method, data can rapidly be obtained from crystals which are smaller than the wavelength of visible light and hence “invisible”. Typically, data sets from multiple nanocrystals are averaged to obtain higher-quality structures. This approach was first demonstrated on an 11-residue peptide derived from the non-amyloid beta component (NAC) region of alpha-synuclein [101]. Other structures solved by this method which are relevant for gaining insight into IDPs include a hexa- and heptapeptide derived from the amyloid core of the Sup35 prion protein [103] and tau-derived peptide VQIVYK [104]. A different X-ray based method, SAXS, is able to provide a degree of insight into the behavior of IDPs in solution. More precisely, the size distribution of proteins in solution can be obtained from this method, providing a measure of the compactness of the solution structure [105].

Multidimensional NMR spectroscopy of IDPs is challenging due to the rapid interconversion between transient conformational states, leading to a severe spectral overlap. The chemical exchange of amide protons with surrounding water molecules is also common and leads to poor signal-to-noise ratios [106]. Performing the experiment at low temperatures can ameliorate this issue to some degree. Alternatively, detection of other NMR-active nuclei (particularly ^13^C and ^15^N) can also overcome the problem of amide proton exchange, and has the benefit of increased chemical shift dispersion compared to ^1^H-NMR [107,108]. Isotopic enrichment is often required for this, and therefore, homonuclear ^13^C-^13^C coupling is a potential issue. In particular, coupling between the carbonyl carbon and amide nitrogen provides information on sequential residues, and is also able to probe prolines, which lack an amide proton altogether.

An elegant NMR-based study was carried out by Veglia et al., who, using a combination of solid-state NMR and solution NMR chemical exchange saturation transfer, studied the interaction of alpha-synuclein with the membranes of synaptic-like vesicles, and showed that the N-terminal, C-terminal, and central NAC regions of this IDP have distinct functions in this interaction [109]. In this work, the N-terminus adopted an alpha-helical structure in these experiments and anchored the protein to the vesicle, while the C-terminus remained largely unstructured. Interestingly, the behavior of the terminal regions was rather insensitive to the lipid composition of the membrane, while the NAC region modulated the interaction strength in a lipid-selective fashion [109]. Given the increased awareness in recent years of the importance of interactions of amyloidogenic disordered peptides and proteins with membranes in several neurodegenerative diseases, this type of work could provide valuable insights into disease mechanisms [110,111,112]. Further information on the use of NMR for the in vitro study of IDPs can be found in the excellent review by Konrat [106]. NMR measurements can also be carried out *in cellula*, which requires the introduction of protein that has been enriched in NMR-active nuclei. This can be accomplished in several ways, for example, the microinjection of exogenously produced protein, or diffusion into the cell through the membrane after treatment with pore-forming toxins. Alternatively, overexpression of proteins of interest can be induced in the cells in which the analysis is to be performed. As this overexpression will outpace normal protein production, transferring the cells to an isotopically-labelled medium at this stage, results in a significant concentration of isotopically-labelled protein of interest. For further information on these methods, the reader is directed to the review by Selenko et al. [113].

Mass spectrometry (MS) offers intriguing avenues for studying the structure of proteins in the gas phase. Electrospray ionization (ESI) is most commonly used for structural studies. Generally speaking, for globular, water-soluble proteins, if they are in their most native-like state immediately prior to the ionization process (in practice, this means ESI from aqueous solutions at physiological pH) this will result in compact, folded protein ions with a narrow distribution of charge states [114]. In contrast to this “native MS” approach, denaturing the protein in solution (e.g., by addition of an organic solvent or lowering the solution pH) results in extended gas-phase conformations with a broad charge state distribution, and possessing higher average charge states than under native conditions. The exact underlying reason for this behavior is still actively being investigated, although it has been proposed that folded and unfolded proteins are transferred into the gas phase through different ESI mechanisms [115]. IDPs possess a relatively extended structure under physiological conditions, and therefore, exhibit a characteristic behavior in MS. Specifically, they consistently generate broad, “non-native-like” charge state distributions under “native MS” conditions [116,117,118,119,120,121,122]. These broad charge state distributions correspond to a tremendous conformational heterogeneity, and it has been shown that IDPs are in fact able to sample an even greater conformational space in the gas phase than in solution [123]. This was accomplished through a combination of molecular dynamics simulations, native MS, and ion mobility (IM) spectrometry [3]. This third technique relies on the gas-phase separation of ions based on their electrophoretic mobility through an inert gas, providing a measure of their rotationally averaged collision cross-section. Recently, Barran et al. investigated the IM-MS behavior of three permutants of the C-terminal IDR of the protein p27^Kip1^, with values for the charge mixing parameter *κ* of 0.14, 0.27 (wild-type), and 0.56. They found that a higher value for *κ* resulted in a more compact gas-phase conformation (smaller collision cross-section) and a shift of the charge state distribution to produce fewer, and lower charge states, consistent with the principles outlined above [124].

Beyond the global (un)folding state from charge state distributions and IM measurements, there are several methods available to obtain structural details through MS. One class of methods involves labelling the protein in the solution in a conformation-sensitive manner, after which the conventional MS/MS analysis maps the number and position of labels. One of the most classic experiments is hydrogen-deuterium exchange [125], in which amide hydrogen atoms are exchanged for deuterium after diluting the protein solution in deuterium oxide. The kinetics of this exchange depend on the protein conformation and dynamics. This creates practical problems for fully unfolded states, as the exchange goes to essentially 100% in milliseconds. However, this method is exquisitely sensitive for probing transient local folding or stabilization, for example, by binding of the IDP to an interaction partner [121,126]. A more direct approach is also possible, in which no solution labelling is performed, but MS/MS is performed of proteins ionized under native-like conditions. For both folded proteins and IDPs, this often results in a fragmentation pattern which is dependent on the protein charge state and conformation, so that structural information can be inferred from this pattern [121,127,128,129].

Single-molecule FRET (Förster/fluorescence resonance energy transfer) is another popular method for structural analysis of IDPs [130,131]. An example was discussed earlier, in which the effect of molecular crowding on the conformational ensemble of IDPs was investigated [57,58]. Essentially, in this technique a protein is labelled at two sites with two different chromophores. This usually requires the introduction of two cysteine residues through mutation and recombinant expression. One of the chromophores, known as the donor, is excited by irradiation at its absorption maximum, and is able to transfer its energy to the second (acceptor) chromophore by dipole-dipole coupling. This then emits a photon at slightly lower energy than that used for excitation of the donor, and this emission is measured. The efficiency of this process drops off quickly as the distance between both chromophores increases (inverse sixth power relation), and therefore, FRET can be used to accurately measure pairwise distances between residues.

A conceptually somewhat similar technique to FRET is tryptophan-cysteine quenching [132]. In this approach, a tryptophan residue is excited to the triplet state using a laser pulse. In the absence of any quenchers, this state has a lifetime of around 40 µs. Cysteine, however, is able to act as an efficient quencher and can induce decay to the singlet ground state in as little as 100 ns. Therefore, the measurement of the lifetime of the excited state can be used to measure the rate of intramolecular contact formation between the tryptophan and cysteine residues [132]. This method has been used by Lapidus et al. to study the aggregation of wild-type alpha-synuclein, as well as several Parkinson’s disease-causing mutants. As this protein lacks both cysteine and tryptophan, a double mutant had to be engineered to enable these experiments [133,134].

## 4. Conclusions and Perspective

Intrinsically disordered proteins defy the conventional structure-function paradigm for how proteins operate, and represent some of the most conformationally dynamic biomolecules known. While they have been described as “*mysterious*” [12] and a “*dark proteome*” [135,136,137] due to the challenge they pose for structural biologists, it is clear that there is order to this chaos, and that their most accurate characterization might be that of “*interaction specialists*” [10]. Due to both their tendency to be involved in many different cellular processes, and the fact that they are balanced on a conformational knife’s edge, mutations in these proteins often have important biomedical implications. This phenomenon has been captured in the D^2^ (“*disorder in disorders*”) concept [138], and makes studying these proteins all the more important. Recent advances in structural biology technology for the condensed state, including the cryo-EM revolution, have made the analysis of these proteins more tractable. Approaches now exist to trap disordered proteins in a particular conformation—often as part of a complex—allowing information to be obtained through, e.g., X-ray crystallography. Meanwhile, mass spectrometry has emerged as a powerful method for IDP analysis in the gas phase, and this analysis is not inherently more difficult than for structured proteins. Undoubtedly, the coming years will see even more studies into the conformational ensembles and dynamics of intrinsically disordered proteins, providing new insights into their many, varied biological roles.
